# E-cadherin gene mutations are rare in adenocarcinomas of the oesophagus

**DOI:** 10.1038/sj.bjc.6690577

**Published:** 1999-07

**Authors:** B P L Wijnhoven, N J de Both, H van Dekken, H W Tilanus, W N M Dinjens

**Affiliations:** The Rotterdam Oesophageal Tumour Study Group, Erasmus University Medical Center Rotterdam, Department of Surgery, University Hospital Dijkzigt, Dr Molewaterplein 40, 3015 GD, Rotterdam, The Netherlands; Department of Pathology, Josephine Nefkens Institute, Dr Molewaterplein 50, 3015 GE, Rotterdam, The Netherlands

**Keywords:** adenocarcinoma oesophagus, E-cadherin, mutations, PCR-SSCP, LOH

## Abstract

Reduced expression of E-cadherin, a cell–cell adhesion molecule, is observed in oesophageal adenocarcinomas and correlates with less favourable pathological parameters and survival. To determine if genetic events lead to reduced E-cadherin expression in these patients, we screened all 16 exons of the E-cadherin gene for mutations with the polymerase chain reaction single-strand conformation polymorphism analysis (PCR-SSCP) technique in 49 resection specimens, including four loco-regional lymph node metastases, four established cell lines and four xenografts. Fifteen exon-spanning primer pairs were used, and in nine amplicons aberrant bands were detected. Sequencing of the amplicons revealed a one base-pair deletion (codon 120; exon 3) in cell lines JROECL 47 and JROECL 50 leading to a premature downstream stop codon. Polymorphisms were identified for amplicons 1, 4/5, 11, 12, 13, 14 and 16 corresponding with data from the literature. Three new polymorphisms were detected for amplicons 2, 3 and 4/5. Loss of heterozygosity (LOH) of the E-cadherin locus on 16q22.1 was examined with four polymorphic markers. LOH was found in 31 of the 48 informative cases (65%). These results show that, despite the frequent LOH of the E-cadherin locus, mutations in the E-cadherin gene are rare events and can not be held responsible for down-regulation of E-cadherin observed in the majority of adenocarcinomas of the oesophagus. © 1999 Cancer Research Campaign


*British Journal of Cancer* (1999) **80**(10), 1652–1657
© 1999 Cancer Research Campaign
Article no. bjoc.1999.0577

### E-cadherin gene mutations are rare in adenocarcinomas of the oesophagus

**BPL Wijnhoven[1], NJ de Both[2], H van Dekken[2], HW Tilanus[1] and WNM Dinjens[2]**

The Rotterdam Oesophageal Tumour Study Group, Erasmus University Medical Center Rotterdam, Department of Surgery, University Hospital Dijkzigt, Dr Molewaterplein 40, 3015 GD, Rotterdam, The Netherlands; [2]Department of Pathology, Josephine Nefkens Institute, Dr Molewaterplein 50, 3015 GE, Rotterdam, The Netherlands

**Summary** Reduced expression of E-cadherin, a cell–cell adhesion molecule, is observed in oesophageal adenocarcinomas and correlates with less favourable pathological parameters and survival. To determine if genetic events lead to reduced E-cadherin expression in these patients, we screened all 16 exons of the E-cadherin gene for mutations with the polymerase chain reaction single-strand conformation polymorphism analysis (PCR-SSCP) technique in 49 resection specimens, including four loco-regional lymph node metastases, four established cell lines and four xenografts. Fifteen exon-spanning primer pairs were used, and in nine amplicons aberrant bands were detected. Sequencing of the amplicons revealed a one base-pair deletion (codon 120; exon 3) in cell lines JROECL 47 and JROECL 50 leading to a premature downstream stop codon. Polymorphisms were identified for amplicons 1, 4/5, 11, 12, 13, 14 and 16 corresponding with data from the literature. Three new polymorphisms were detected for amplicons 2, 3 and 4/5. Loss of heterozygosity (LOH) of the E-cadherin locus on 16q22.1 was examined with four polymorphic markers. LOH was found in 31 of the 48 informative cases (65%). These results show that, despite the frequent LOH of the E-cadherin locus, mutations in the E-cadherin gene are rare events and can not be held responsible for down-regulation of E-cadherin observed in the majority of adenocarcinomas of the oesophagus.

**Keywords**: adenocarcinoma oesophagus; E-cadherin; mutations; PCR-SSCP; LOH

Cadherins are a family of $Ca^{2+}$-dependent cell–cell adhesion molecules. E-cadherin is a transmembrane protein with five tandemly repeated extracellular domains and a cytoplasmic domain that connects to the actin cytoskeleton through a complex with α-, β- and γ-catenins. E-cadherin is expressed on the cell surface in most epithelial tissues and is important for establishing cell polarity, maintaining epithelial integrity and cellular differentiation (Takeichi, 1991). The potential for E-cadherin to serve as an invasion or metastatic suppressor in epithelial tumorigenesis has been elucidated from in vitro studies. E-cadherin-negative epithelial cells grow invasive with a mesenchymal phenotype. After transfection with E-cadherin cDNA, epithelial structure is restored (Frixen et al, 1991; Vleminckx et al, 1991).

Consistent with this observation is a reduced or absent E-cadherin expression in various epithelial cancers showing invasive growth (Gabbert et al, 1996; Kuczyk et al, 1998; Sulzer et al, 1998). Decreased expression of E-cadherin in Barrett's oesophagus, adenocarcinomas of the oesophagus and gastro-oesophageal junction was found to be related with progression of Barrett's oesophagus to adenocarcinoma, tumour stage and lymph node metastasis (Bongiorno et al, 1995; Bailey et al, 1998). Furthermore, E-cadherin expression was an independent variable predicting survival in patients after resection for adenocarcinomas of the oesophagus (Krishnadath et al, 1997).

*Received 3 September 1998*
*Revised 12 January 1999*
*Accepted 27 January 1999*

*Correspondence to:* WNM Dinjens

Loss of E-cadherin expression resulted in the transition from well-differentiated adenoma to invasive carcinoma in mouse pancreatic β-cell carcinogenesis (Perl et al, 1998). Inactivating mutations in the E-cadherin gene have been described for various tumours such as lobular breast cancer (in situ) and diffuse infiltrating gastric cancer concomitant with loss of heterozygosity (LOH) at the E-cadherin locus in E-cadherin-negative tumours (Becker and Hofler, 1995; Berx et al, 1995, 1996; Vos et al, 1997). Moreover, two recent studies showed that germline mutations in the E-cadherin gene are associated with early onset familial gastric cancer (Gayther et al, 1998; Guilford et al, 1998). Whether genetic alterations in the E-cadherin gene play a role in the pathogenesis of adenocarcinomas of the oesophagus and gastro-oesophageal junction is not known. Therefore, we screened adenocarcinomas of the oesophagus and gastro-oesophageal junction for E-cadherin gene mutations and LOH.

### METHODS

### Tumour specimens

Fresh samples of adenocarcinomas of the distal oesophagus or gastro-oesophageal junction were obtained from 45 resection specimens. For analysis the tumour samples were microdissected to enrich for cancer cells (> 75%). Nineteen tumours showed microscopic evidence of surrounding intestinal metaplasia indicative for Barrett's carcinomas. Samples of tumour and normal gastric epithelium or squamous epithelium of the oesophagus were snap-frozen and stored in liquid nitrogen. Four lymph nodes infiltrated by tumour were frozen as well.

### Cell lines and xenografts

In vitro cell lines form three adenocarcinomas JROECL19, JROECL33, JROECL50 and one adenosquamous carcinoma JROECL47 established by Rockett et al (1997) were obtained from European Collection of Cell Cultures (ECACC). In vivo xenografts were obtained after transplantation of tumour tissue to female nude mice, 4–6 weeks of age. From three lymph node metastases M2.1X1, M9X1, M4.1X2 and one primary tumour P23X1, xenografts were obtained.

### DNA preparation

DNA from cell lines was isolated according to standard procedures. Genomic DNA from xenografts and tumour samples and normal tissue was isolated from consecutive 5-µm cryostat tissue sections by overnight proteinase K incubation at 55°C followed by phenol extraction and ethanol precipitation. DNA pellets were dissolved in TE (10 mM Tris–HCl, pH 7.8; 1 mM EDTA). The tumour tissue samples contained at least 75% tumour cells.

### Analysis of the E-cadherin gene by PCR-SSCP

The entire open reading frame of the E-cadherin gene was screened for mutations using 15 exon-spanning primer pairs (Berx et al, 1995). Genomic DNA was used at 50–100 ng per 15 µl reaction mixture containing 1.5 mM magnesium chloride, 0.02 mM dATP, 0.2 mM dGTP, dTTP and dCTP each, 0.8 µCi $\alpha$-$^{32}$PdATP (Amersham, Buckinghamshire, UK), 20 pmol of each primer and 0.2 U *Taq* polymerase (Promega, Madison, WI, USA). Each PCR was overlaid with mineral oil. PCR was performed for 35 cycles (denaturing at 95°C for 30 s, annealing at the appropriate temperature for 45 s and extension at 72°C for 1 min). A final extension step was carried out at 72°C for 10 min. PCR products were diluted 1:4 with a loading buffer (95% formamide, 10 mM EDTA (pH 8.0), 0.025% bromophenol blue and 0.025% xylene cyanol), denatured at 95°C for 4 min and snap-cooled on ice. Appropriate aliquots of the radiolabelled PCR products were separated on a non-denaturing polyacrylamide gel (6% polyacrylamide) containing 10% glycerol and run at 7 W overnight at room temperature in 1 × TBE running buffer. Gels were fixed in acetic acid (10%), dried on blotting paper (Schleicher & Schuell, Dassel, Germany) on a vacuum gel dryer and exposed to X-ray film overnight at –70°C, using intensifying screens. DNA with aberrantly migrating PCR-SSCP fragments was reamplified and purified over QIAquick spin columns (Qiagen, Hilden, Germany), cloned into a PGEM-T easy vector (Promega, Madison, WI, USA), and sequenced with $\alpha$-$^{35}$SdATP according to the dideoxy chain termination method. Electrophoresis of the sequenced samples was carried out on an 8% denaturing polyacrylamide gel. After fixation and drying, gels were exposed to X-ray film for 1–3 days at room temperature.

### LOH determination

In 51 tumours LOH was determined with microsatellite markers that map on 16q22.1 where the E-cadherin gene is located. Markers tested were: D16S503, D16S265, D16S398 and D16S512. Markers were tested on 100 ng of tumour and normal DNA in a PCR reaction as described previously (Trapman et al, 1994). LOH was established by visual comparison of the intensity of allelic bands obtained from tumour samples with those from normal DNAs.

### RESULTS

### PCR-SSCP

All 16 exons and exon–intron boundaries of the E-cadherin gene were analysed for genetic alterations in four cell lines, four xenografts and 49 tumour samples (45 primary tumours and four corresponding locoregional lymph node metastases). In total, 37 aberrant bands were detected throughout nine different amplicons. Cell line, xenograft and tumour DNA with aberrant SSCP patterns were compared with the amplification products of patient matched non-tumorous DNA. In most tumours the aberrant SSCP pattern was also present in the corresponding normal DNA (Figure 1). A tumour-restricted mobility shift was found in cell lines JROECL47 and JROECL50 in amplicon 3 (Figure 1B). Upon sequencing a one base-pair deletion in codon 120 (exon 3) was detected (Figure 2D and Table 1). Both cell lines were derived from the same primary tumour (SJ Darnton, personal communication), in which this mutation could not be detected. Sequence analysis of all the other SSCP band shifts revealed eight known polymorphisms and three new genetic alterations (Figure 2 A–C and Table 2). Furthermore, upon sequencing of amplicon 16 a discrepancy with the published sequence of intron 15 of the E-cadherin gene deposited in EMBL/GenBank database libraries (accession no. Z13009) was detected (5′ intron15- … ctt**g**ag-3′ exon 16).

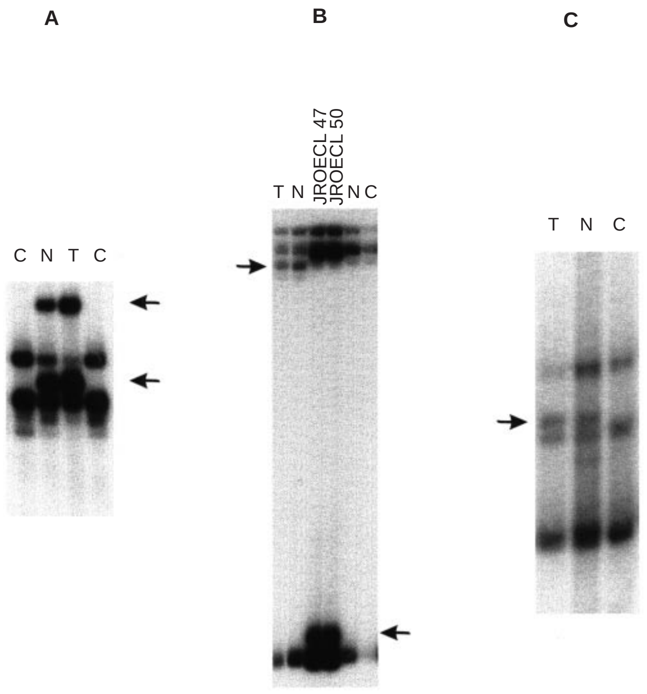

**Figure 1** PCR-SSCP analysis of E-cadherin amplicons 2 (**A**), 3 (**B**) and 4/5 (**C**) from three oesophageal adenocarcinoma specimens and two cell lines JROECL 47 and JROECL 50. Mobility shifts are identified by arrows. C, control DNA; T, tumour DNA; N, corresponding non-tumourous DNA

*© 1999 Cancer Research Campaign*

**Table 1** Tumour-restricted mutation in cell lines JROECL 47 and JROECL 50[a]

| Amplicon | Site | Nucleotide change |
|---|---|---|
| 3 | Cdn 120 | Deletion G: frameshift leading to stopcodon at codon 214 in exon 5 |

[a]Two cell lines derived from a primary tumour of one patient.

### LOH determination

In Figure 3 examples are presented of the analysis of the four microsatellite loci in different tumour DNAs which showed allelic loss. Of the 48 informative cases, 31 (65%) showed LOH of at least one marker (Figure 4).

### DISCUSSION

Reduced expression of E-cadherin is frequently seen in the majority of adenocarcinomas of the oesophagus and gastro-oesophageal junction with or without Barrett's epithelium (Bongiorno et al, 1995; Swami et al, 1995; Bailey et al, 1998). However, E-cadherin immunoreactivity does not provide information about the function of the protein and gene integrity. For example, mutations in the E-cadherin gene leading to loss of adhesive potential but with normal cellular localization of the protein have been described (Becker et al, 1994; Oda et al, 1994). In this study we have analysed if genetic alterations in the E-cadherin gene are involved in the reduced expression of E-cadherin in adenocarcinomas of the oesophagus and gastro-oesophageal junction. In an earlier study, eight out of 49 tumours were analysed for expression of E-cadherin by means of immunohistochemistry. Seven out of eight of these adenocarcinomas showed reduced expression for E-cadherin (Krishnadath et al, 1997).

In our series of 57 adenocarcinomas of the oesophagus only one tumour restricted DNA alteration in the E-cadherin gene was detected. In two cell lines JROECL 47 and JROECL 50, derived from the same tumour, a one base-pair deletion was found leading to a premature stopcodon resulting in a truncated protein lacking

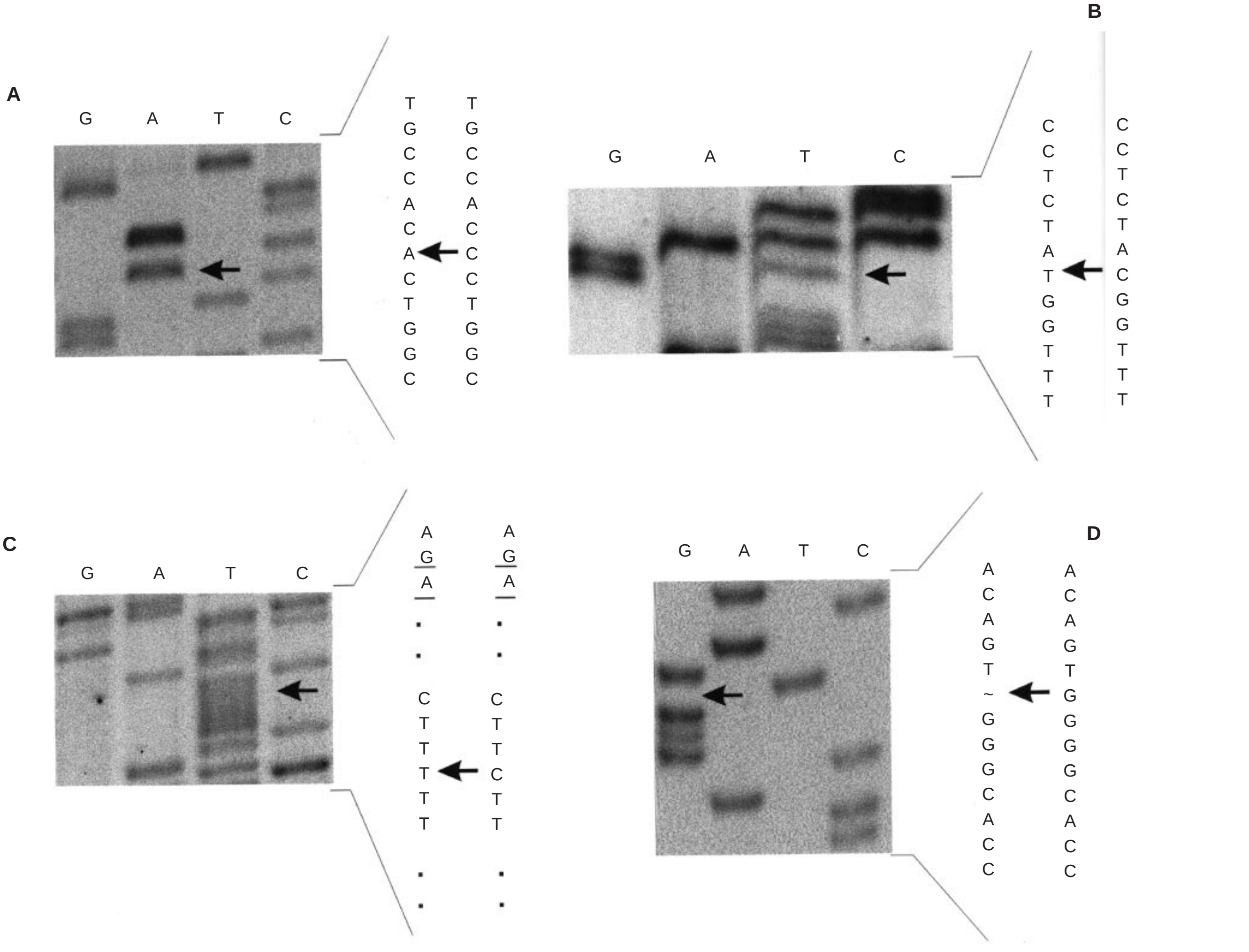

**Figure 2** Sequence analysis for the three tumours and cell line JROECL 47 with abnormally shifted PCR-SSCP bands as shown in Figure 1. Mutated sequence (left) and wild-type sequence (right) are shown at the right of each figure. Base-pair alterations are indicated by arrows. Splice recognition site is shown by the underlined characters in Figure 2C. For exon 2 (**A**) and exon 3 (**B**) we identified mis-sense mutations, also present in the normal control DNA of these patients. For amplicon 4/5 a base-pair change was observed in intron 4 (**C**). One frameshift mutation caused by a 1-bp deletion was detected for exon 3 (**D**) in the two cell lines JROECL 47 and JROECL 50

 *© 1999 Cancer Research Campaign*

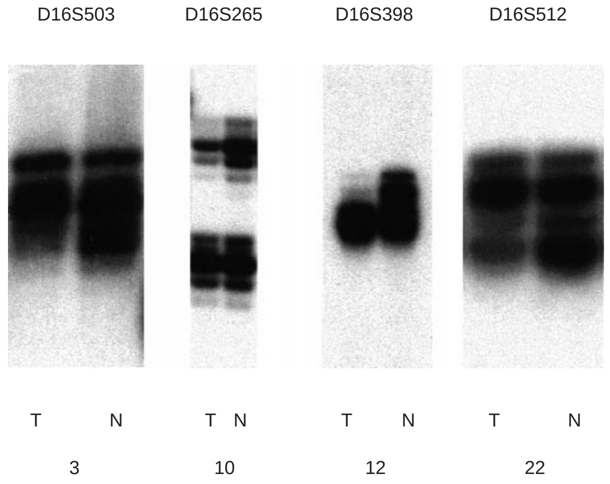

**Figure 3** Loss of heterozygosity in tumour 3 (D16S503), 10 (D16S265), 12 (D16S398) and 22 (D16S512). Loss of one allele is visible in the lane containing tumour DNA (T) compared with the lane containing normal DNA (N)

part of the extracellular binding region as well as the transmembrane and intracellular domain. So far, this mutation has not been reported in the literature. Both cell lines still have a non-mutated E-cadherin allele as demonstrated by the heterozygous exon 3 SSCP pattern indicating two different alleles. We were unable to demonstrate this mutation in the primary tumour. Obviously, the mutation was present in a minor subpopulation of the primary tumour or has developed during establishment of both cell lines.

PCR-SSCP analysis is a robust mutation detection procedure (Orita et al, 1989; Sheffield et al, 1993), indicated by the finding of eight known polymorphisms and three not yet described DNA alterations. However, we cannot rule out the possibility that mutations remained undetected by this procedure. All three new DNA alterations are also present in the patients' normal DNA. Two of these alterations lead to amino acid substitutions in the precursor sequence of the E-cadherin gene. Recently, germline mutations leading to truncated E-cadherin were identified in familial gastric cancer in New Zealand and Europe (Gayther et al, 1998; Guilford et al, 1998). Because no loss of the normal E-cadherin allele in the tumours occurred we consider these DNA alterations as polymorphisms. Furthermore, we have no evidence of a familial predisposition to oesophageal cancer in any of our patients.

Allelic loss at the E-cadherin gene locus 16q22.1 has been reported in 30–50% of breast, prostate and hepatocellular cancer (Tamura, 1997). Our data show LOH of this locus in two-thirds of the oesophageal adenocarcinomas. But the high percentage of 16q22.1 LOH without concomitant mutation of the remaining E-cadherin gene might point to another tumour suppressor gene on 16q involved in the genesis or progression of oesophageal adenocarcinomas. We cannot rule out the possibility that homozygous deletions are present in these carcinomas. Especially in DNA derived from resection specimens homozygous deletions can escape detection due to the contamination with non-tumorous DNA. However, all exons could be amplified with DNA derived from the cell lines and xenografts with exon spanning primers, which makes it unlikely that homozygous deletions have occurred.

Alternatively, if the high frequency of LOH at 16q22.1 truly reflects loss of one E-cadherin allele, this could point in the

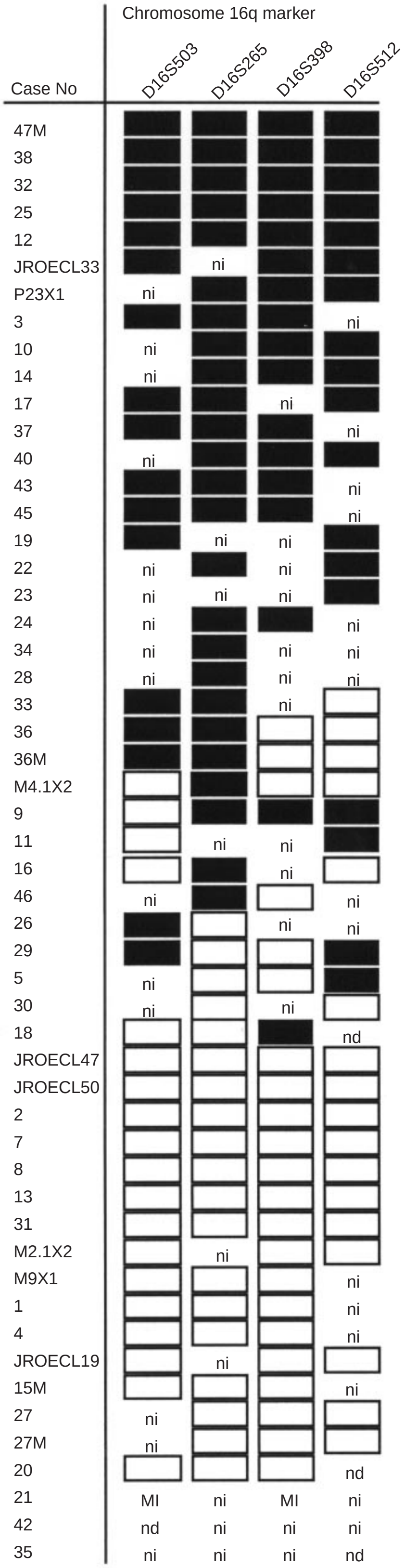

**Figure 4** Patterns of allelic loss in oesophageal adenocarcinomas at the E-cadherin locus on chromosome 16q22.1. Closed rectangle, loss of heterozygosity; white rectangle, heterozygosity retained; ni, non-informative; MI, microsatellite instability; nd, no data obtainable. Note that 36M and 27M are lymph node metastases from primary tumours 36 and 27 respectively

© 1999 Cancer Research Campaign

**Table 2** Polymorphisms identified in the E-cadherin gene

| Amplicon | Site | Nucleotide change | Observed frequency[a] (57 tumours) | Literature[b] |
|---|---|---|---|---|
| 1 | Non-coding (5′ UTR) | TCC→TGC | 2/57 | 1 |
| 2 | Cdn 30 | CCT→ACT *Pro→Thr* | 1/57 | Unknown |
| 3 | Cdn 90 | CGG→TGG Arg→Trp | 1/57 | Unknown |
| 4/5 | Intron 4 | gtagagaaa**g**→..a**c** | 2/57 | 1 |
| | Intron 4 | C→T 18 nt upstream exon 5 | 1/57 | Unknown |
| 11 | Cdn 560 | ACG→ACC *Thr→Thr* | 2/57 | 1, 2 |
| 12 | Cdn 632 | CAC→CAT *His→His* | 1/57 | 1, 2, 3 |
| 13 | Cdn 692 | GCT→GCC *Ala→Ala* | 14/57 | 1, 2, 4 |
| | Intron 12 | T→C | 12/57 | 1, 4 |
| 14 | Cdn 751 | AAC→AAT *Asn→Lys* | 1/57 | 1, 2 |
| 16 | Cdn 878 | GGC→GGT *Gly→Gly* | 1/57 | 3 |

[a]Splice recognition site is shown by the underlined characters. [b]Polymorphisms also reported by [1]Berx et al, 1996; [2]Guilford et al, 1998; [3]Risinger et al, 1994; [4]Soares et al, 1997.

direction of gene dosage effects as formerly proposed on the basis of experimental studies (Vleminckx et al, 1991). However, a previous study by Ilyas et al could not establish a correlation between allelic loss and immunohistochemical E-cadherin expression in colorectal cancers (Ilyas et al, 1997). Besides loss of one E-cadherin allele, other mechanisms leading to down-regulation of E-cadherin in oesophageal adenocarcinomas could also be involved.

Hypermethylation of the 5′ CpG islands within the proximal promoter region of the E-cadherin gene has been found responsible for (temporary) down-regulation of E-cadherin in several cancers (Graff et al, 1998; Hiraguri et al, 1998; Saito et al, 1998). However, not only E-cadherin but also other members of the junctional complex have been shown to play a role in tumorigenesis. In several tumour cell lines showing impaired cell–cell adhesion and reduced expression of E-cadherin, mutations in α-catenin and β-catenin were identified (Shimoyama et al, 1992; Kawanishi et al, 1995). A cytoplasmic and nuclear staining pattern of β-catenin has been observed in adenocarcinomas of the oesophagus in cases in which E-cadherin was reduced (Krishnadath et al, 1997; Bailey et al, 1998). These tumours could harbour mutated β-catenin, as was recently described for colon carcinomas and hepatocellular carcinomas (de La Coste et al, 1998; Iwao et al, 1998). Whether or not mutations in the catenins play a role in the pathogenesis of oesophageal cancer is presently unknown.

In conclusion, our results show that E-cadherin gene mutations are not involved in the subsequent progression of Barrett's epithelium to dysplasia and to adenocarcinoma of the oesophagus. Whether LOH at the E-cadherin locus contributes to the heterogenous expression of E-cadherin remains to be determined.

### ACKNOWLEDGEMENTS

The authors thank Dr S.J. Darnton (Birmingham Heartlands Hospital, Birmingham, UK) for providing tissue blocks for the patients from whom the cell lines were derived. H. Sleddens and N. Groen are acknowledged for technical assistance. This work was supported by Gastrostart from the Dutch Society of Gastroenterology.

### REFERENCES

Bailey T, Biddlestone L, Shepherd N, Barr H, Warner P and Jankowski J (1998) Altered cadherin and catenin complexes in the Barrett's esophagus–dysplasia–adenocarcinoma sequence: correlation with disease progression and dedifferentiation. *Am J Pathol* **152**: 135–144

Becker KF and Hofler H (1995) Frequent somatic allelic inactivation of the E-cadherin gene in gastric carcinomas. *J Natl Cancer Inst* **87**: 1082–1084

Becker KF, Atkinson MJ, Reich U, Becker I, Nekarda H, Siewert JR and Hofler H (1994) E-cadherin gene mutations provide clues to diffuse type gastric carcinomas. *Cancer Res* **54**: 3845–3852

Berx G, Cleton-Jansen AM, Nollet F, de Leeuw WJ, van de Vijver M, Cornelisse C and van Roy F (1995) E-cadherin is a tumour/invasion suppressor gene mutated in human lobular breast cancers. *Embo J* **14**: 6107–6115

Berx G, Cleton-Jansen AM, Strumane K, de Leeuw WJ, Nollet F, van Roy F and Cornelisse C (1996) E-cadherin is inactivated in a majority of invasive human lobular breast cancers by truncation mutations throughout its extracellular domain. *Oncogene* **13**: 1919–1925

Bongiorno PF, al-Kasspooles M, Lee SW, Rachwal WJ, Moore JH, Whyte RI, Orringer MB and Beer DG (1995) E-cadherin expression in primary and metastatic thoracic neoplasms and in Barrett's oesophagus. *Br J Cancer* **71**: 166–172

de La Coste A, Romagnolo B, Billuart P, Renard CA, Buendia MA, Soubrane O, Fabre M, Chelly J, Beldjord C, Kahn A and Perret C (1998) Somatic mutations of the beta-catenin gene are frequent in mouse and human hepatocellular carcinomas. *Proc Natl Acad Sci USA* **95**: 8847–8851

Frixen UH, Behrens J, Sachs M, Eberle G, Voss B, Warda A, Lochner D and Birchmeier W (1991) E-cadherin-mediated cell–cell adhesion prevents invasiveness of human carcinoma cells. *J Cell Biol* **113**: 173–185

Gabbert HE, Mueller W, Schneiders A, Meier S, Moll R, Birchmeier W and Hommel G (1996) Prognostic value of E-cadherin expression in 413 gastric carcinomas. *Int J Cancer* **69**: 184–189

Gayther SA, Gorringe KL, Ramus SJ, Huntsman D, Roviello F, Grehan N, Machado JC, Pinto E, Seruca R, Halling K, MacLeod P, Powell SM, Jackson CE, Ponder

*© 1999 Cancer Research Campaign*

BAJ and Caldas C (1998) Identification of germ-line E-cadherin mutations in gastric cancer families of European origin. *Cancer Res* **58**: 4086–4089

Graff JR, Greenberg VE, Herman JG, Westra WH, Boghaert ER, Ain KB, Saji M, Zeiger MA, Zimmer SG and Baylin SB (1998) Distinct patterns of E-cadherin CpG island methylation in papillary, follicular, Hurthle's cell, and poorly differentiated human thyroid carcinoma. *Cancer Res* **58**: 2063–2066

Guilford P, Hopkins J, Harraway J, McLeod M, McLeod N, Harawira P, Taite H, Scoular R, Miller A and Reeve AE (1998) E-cadherin germline mutations in familial gastric cancer. *Nature* **392**: 402–405

Hiraguri S, Godfrey T, Nakamura H, Graff J, Collins C, Shayesteh L, Doggett N, Johnson K, Wheelock M, Herman J, Baylin S, Pinkel D and Gray J (1998) Mechanisms of inactivation of E-cadherin in breast cancer cell lines. *Cancer Res* **58**: 1972–1977

Ilyas M, Tomlinson IP, Hanby A, Talbot IC and Bodmer WF (1997) Allele loss, replication errors and loss of expression of E-cadherin in colorectal cancers. *Gut* **40**: 654–659

Iwao K, Nakamori S, Kameyama M, Imaoka S, Kinoshita M, Fukui T, Ishiguro S, Nakamura Y and Miyoshi Y (1998) Activation of the beta-catenin gene by interstitial deletions involving exon 3 in primary colorectal carcinomas without adenomatous polyposis coli mutations. *Cancer Res* **58**: 1021–1026

Kawanishi J, Kato J, Sasaki K, Fujii S, Watanabe N and Niitsu Y (1995) Loss of E-cadherin-dependent cell–cell adhesion due to mutation of the beta-catenin gene in a human cancer cell line, HSC-39. *Mol Cell Biol* **15**: 1175–1181

Krishnadath KK, Tilanus HW, van Blankenstein M, Hop WC, Kremers ED, Dinjens WN and Bosman FT (1997) Reduced expression of the cadherin–catenin complex in oesophageal adenocarcinoma correlates with poor prognosis. *J Pathol* **182**: 331–338

Kuczyk M, Serth J, Machtens S, Bokemeyer C, Bathke W, Stief C and Jonas U (1998) Expression of E-cadherin in primary prostate cancer: correlation with clinical features [In Process Citation]. *Br J Urol* **81**: 406–412

Oda T, Kanai Y, Oyama T, Yoshiura K, Shimoyama Y, Birchmeier W, Sugimura T and Hirohashi S (1994) E-cadherin gene mutations in human gastric carcinoma cell lines. *Proc Natl Acad Sci USA* **91**: 1858–1862

Orita M, Iwahana H, Kanazawa H, Hayashi K and Sekiya T (1989) Detection of polymorphisms of human DNA by gel electrophoresis as single-strand conformation polymorphisms. *Proc Natl Acad Sci USA* **86**: 2766–2770

Perl AK, Wilgenbus P, Dahl U, Semb H and Christofori G (1998) A causal role for E-cadherin in the transition from adenoma to carcinoma. *Nature* **392**: 190–193

Risinger JI, Berchuck A, Kohler MF and Boyd J (1994) Mutations of the E-cadherin gene in human gynecologic cancers. *Nat Genet* **7**: 98–102

Rockett JC, Larkin K, Darnton SJ, Morris AG and Matthews HR (1997) Five newly established oesophageal carcinoma cell lines: phenotypic and immunological characterization. *Br J Cancer* **75**: 258–263

Saito Y, Takazawa H, Uzawa K, Tanzawa H and Sato K (1998) Reduced expression of E-cadherin in oral squamous cell carcinoma: relationship with DNA methylation of 5′ CpG island. *Int J Oncol* **12**: 293–298

Sheffield VC, Beck JS, Kwitek AE, Sandstrom DW and Stone EM (1993) The sensitivity of single-strand conformation polymorphism analysis for the detection of single base substitutions. *Genomics* **16**: 325–332

Shimoyama Y, Nagafuchi A, Fujita S, Gotoh M, Takeichi M, Tsukita S and Hirohashi S (1992) Cadherin dysfunction in a human cancer cell line: possible involvement of loss of alpha-catenin expression in reduced cell–cell adhesiveness. *Cancer Res* **52**: 5770–5774

Soares P, Berx G, van Roy F and Sobrinho-Simoes M (1997) E-cadherin gene alterations are rare events in thyroid tumors. *Int J Cancer* **70**: 32–38

Sulzer MA, Leers MP, van Noord JA, Bollen EC and Theunissen PH (1998) Reduced E-cadherin expression is associated with increased lymph node metastasis and unfavorable prognosis in non-small cell lung cancer. *Am J Respir Crit Care Med* **157**: 1319–1323

Swami S, Kumble S and Triadafilopoulos G (1995) E-cadherin expression in gastroesophageal reflux disease, Barrett's esophagus, and esophageal adenocarcinoma: an immunohistochemical and immunoblot study. *Am J Gastroenterol* **90**: 1808–1813

Takeichi M (1991) Cadherin cell adhesion receptors as a morphogenetic regulator. *Science* **251**: 1451–1455

Tamura S (1997) The E-cadherin-mediated cell–cell adhesion system in human cancers. *Br J Surg* **84**: 899–900

Trapman J, Sleddens HF, van der Weiden MM, Dinjens WN, Konig JJ, Schroder FH, Faber PW and Bosman FT (1994) Loss of heterozygosity of chromosome 8 microsatellite loci implicates a candidate tumor suppressor gene between the loci D8S87 and D8S133 in human prostate cancer. *Cancer Res* **54**: 6061–6064

Vleminckx K, Vakaet L, Jr, Mareel M, Fiers W and van Roy F (1991) Genetic manipulation of E-cadherin expression by epithelial tumor cells reveals an invasion suppressor role. *Cell* **66**: 107–119

Vos CB, Cleton-Jansen AM, Berx G, de Leeuw WJ, ter Haar NT, van Roy F, Cornelisse CJ, Peterse JL and van de Vijver MJ (1997) E-cadherin inactivation in lobular carcinoma in situ of the breast: an early event in tumorigenesis. *Br J Cancer* **76**: 1131–1133

© 1999 Cancer Research Campaign

## References

[PDF_00525] Bailey T., Biddlestone L., Shepherd N., Barr H., Warner P., Jankowski J. (1998). Altered cadherin and catenin complexes in the Barrett's esophagus-dysplasia-adenocarcinoma sequence: correlation with disease progression and dedifferentiation.. Am J Pathol.

[PDF_00531] Becker K. F., Atkinson M. J., Reich U., Becker I., Nekarda H., Siewert J. R., Höfler H. (1994). E-cadherin gene mutations provide clues to diffuse type gastric carcinomas.. Cancer Res.

[PDF_00529] Becker K. F., Höfler H. (1995). Frequent somatic allelic inactivation of the E-cadherin gene in gastric carcinomas.. J Natl Cancer Inst.

[PDF_00534] Berx G., Cleton-Jansen A. M., Nollet F., de Leeuw W. J., van de Vijver M., Cornelisse C., van Roy F. (1995). E-cadherin is a tumour/invasion suppressor gene mutated in human lobular breast cancers.. EMBO J.

[PDF_00537] Berx G., Cleton-Jansen A. M., Strumane K., de Leeuw W. J., Nollet F., van Roy F., Cornelisse C. (1996). E-cadherin is inactivated in a majority of invasive human lobular breast cancers by truncation mutations throughout its extracellular domain.. Oncogene.

[PDF_00542] Bongiorno P. F., al-Kasspooles M., Lee S. W., Rachwal W. J., Moore J. H., Whyte R. I., Orringer M. B., Beer D. G. (1995). E-cadherin expression in primary and metastatic thoracic neoplasms and in Barrett's oesophagus.. Br J Cancer.

[PDF_00549] Frixen U. H., Behrens J., Sachs M., Eberle G., Voss B., Warda A., Löchner D., Birchmeier W. (1991). E-cadherin-mediated cell-cell adhesion prevents invasiveness of human carcinoma cells.. J Cell Biol.

[PDF_00552] Gabbert H. E., Mueller W., Schneiders A., Meier S., Moll R., Birchmeier W., Hommel G. (1996). Prognostic value of E-cadherin expression in 413 gastric carcinomas.. Int J Cancer.

[PDF_00593] Gayther S. A., Gorringe K. L., Ramus S. J., Huntsman D., Roviello F., Grehan N., Machado J. C., Pinto E., Seruca R., Halling K. (1998). Identification of germ-line E-cadherin mutations in gastric cancer families of European origin.. Cancer Res.

[PDF_00595] Graff J. R., Greenberg V. E., Herman J. G., Westra W. H., Boghaert E. R., Ain K. B., Saji M., Zeiger M. A., Zimmer S. G., Baylin S. B. (1998). Distinct patterns of E-cadherin CpG island methylation in papillary, follicular, Hurthle's cell, and poorly differentiated human thyroid carcinoma.. Cancer Res.

[PDF_00599] Guilford P., Hopkins J., Harraway J., McLeod M., McLeod N., Harawira P., Taite H., Scoular R., Miller A., Reeve A. E. (1998). E-cadherin germline mutations in familial gastric cancer.. Nature.

[PDF_00602] Hiraguri S., Godfrey T., Nakamura H., Graff J., Collins C., Shayesteh L., Doggett N., Johnson K., Wheelock M., Herman J. (1998). Mechanisms of inactivation of E-cadherin in breast cancer cell lines.. Cancer Res.

[PDF_00606] Ilyas M., Tomlinson I. P., Hanby A., Talbot I. C., Bodmer W. F. (1997). Allele loss, replication errors and loss of expression of E-cadherin in colorectal cancers.. Gut.

[PDF_00609] Iwao K., Nakamori S., Kameyama M., Imaoka S., Kinoshita M., Fukui T., Ishiguro S., Nakamura Y., Miyoshi Y. (1998). Activation of the beta-catenin gene by interstitial deletions involving exon 3 in primary colorectal carcinomas without adenomatous polyposis coli mutations.. Cancer Res.

[PDF_00613] Kawanishi J., Kato J., Sasaki K., Fujii S., Watanabe N., Niitsu Y. (1995). Loss of E-cadherin-dependent cell-cell adhesion due to mutation of the beta-catenin gene in a human cancer cell line, HSC-39.. Mol Cell Biol.

[PDF_00616] Krishnadath K. K., Tilanus H. W., van Blankenstein M., Hop W. C., Kremers E. D., Dinjens W. N., Bosman F. T. (1997). Reduced expression of the cadherin-catenin complex in oesophageal adenocarcinoma correlates with poor prognosis.. J Pathol.

[PDF_00620] Kuczyk M., Serth J., Machtens S., Bokemeyer C., Bathke W., Stief C., Jonas U. (1998). Expression of E-cadherin in primary prostate cancer: correlation with clinical features.. Br J Urol.

[PDF_00623] Oda T., Kanai Y., Oyama T., Yoshiura K., Shimoyama Y., Birchmeier W., Sugimura T., Hirohashi S. (1994). E-cadherin gene mutations in human gastric carcinoma cell lines.. Proc Natl Acad Sci U S A.

[PDF_00626] Orita M., Iwahana H., Kanazawa H., Hayashi K., Sekiya T. (1989). Detection of polymorphisms of human DNA by gel electrophoresis as single-strand conformation polymorphisms.. Proc Natl Acad Sci U S A.

[PDF_00629] Perl A. K., Wilgenbus P., Dahl U., Semb H., Christofori G. (1998). A causal role for E-cadherin in the transition from adenoma to carcinoma.. Nature.

[PDF_00631] Risinger J. I., Berchuck A., Kohler M. F., Boyd J. (1994). Mutations of the E-cadherin gene in human gynecologic cancers.. Nat Genet.

[PDF_00633] Rockett J. C., Larkin K., Darnton S. J., Morris A. G., Matthews H. R. (1997). Five newly established oesophageal carcinoma cell lines: phenotypic and immunological characterization.. Br J Cancer.

[PDF_00636] Saito Y., Takazawa H., Uzawa K., Tanzawa H., Sato K. (1998). Reduced expression of E-cadherin in oral squamous cell carcinoma: relationship with DNA methylation of 5' CpG island.. Int J Oncol.

[PDF_00639] Sheffield V. C., Beck J. S., Kwitek A. E., Sandstrom D. W., Stone E. M. (1993). The sensitivity of single-strand conformation polymorphism analysis for the detection of single base substitutions.. Genomics.

[PDF_00642] Shimoyama Y., Nagafuchi A., Fujita S., Gotoh M., Takeichi M., Tsukita S., Hirohashi S. (1992). Cadherin dysfunction in a human cancer cell line: possible involvement of loss of alpha-catenin expression in reduced cell-cell adhesiveness.. Cancer Res.

[PDF_00646] Soares P., Berx G., van Roy F., Sobrinho-Simões M. (1997). E-cadherin gene alterations are rare events in thyroid tumors.. Int J Cancer.

[PDF_00648] Sulzer M. A., Leers M. P., van Noord J. A., Bollen E. C., Theunissen P. H. (1998). Reduced E-cadherin expression is associated with increased lymph node metastasis and unfavorable prognosis in non-small cell lung cancer.. Am J Respir Crit Care Med.

[PDF_00652] Swami S., Kumble S., Triadafilopoulos G. (1995). E-cadherin expression in gastroesophageal reflux disease, Barrett's esophagus, and esophageal adenocarcinoma: an immunohistochemical and immunoblot study.. Am J Gastroenterol.

[PDF_00656] Takeichi M. (1991). Cadherin cell adhesion receptors as a morphogenetic regulator.. Science.

[PDF_00658] Tamura S. (1997). The E-cadherin-mediated cell-cell adhesion system in human cancers.. Br J Surg.

[PDF_00660] Trapman J., Sleddens H. F., van der Weiden M. M., Dinjens W. N., Konig J. J., Schroder F. H., Faber P. W., Bosman F. T. (1994). Loss of heterozygosity of chromosome 8 microsatellite loci implicates a candidate tumor suppressor gene between the loci D8S87 and D8S133 in human prostate cancer.. Cancer Res.

[PDF_00664] Vleminckx K., Vakaet L., Mareel M., Fiers W., van Roy F. (1991). Genetic manipulation of E-cadherin expression by epithelial tumor cells reveals an invasion suppressor role.. Cell.

[PDF_00668] Vos C. B., Cleton-Jansen A. M., Berx G., de Leeuw W. J., ter Haar N. T., van Roy F., Cornelisse C. J., Peterse J. L., van de Vijver M. J. (1997). E-cadherin inactivation in lobular carcinoma in situ of the breast: an early event in tumorigenesis.. Br J Cancer.

[PDF_00545] de La Coste A., Romagnolo B., Billuart P., Renard C. A., Buendia M. A., Soubrane O., Fabre M., Chelly J., Beldjord C., Kahn A. (1998). Somatic mutations of the beta-catenin gene are frequent in mouse and human hepatocellular carcinomas.. Proc Natl Acad Sci U S A.

